# Role of chymase in cigarette smoke-induced pulmonary artery remodeling and pulmonary hypertension in hamsters

**DOI:** 10.1186/1465-9921-11-36

**Published:** 2010-03-31

**Authors:** Tao Wang, Su-Xia Han, Shang-Fu Zhang, Yun-Ye Ning, Lei Chen, Ya-Juan Chen, Guang-Ming He, Dan Xu, Jin An, Ting Yang, Xiao-Hong Zhang, Fu-Qiang Wen

**Affiliations:** 1 Division of Pulmonary Diseases, State Key Laboratory of Biotherapy of China, and Department of Respiratory Medicine, West China Hospital of Sichuan University, Chengdu, Sichuan 610041, PR China; 2 Department of Cardiology, Fifth Affiliated Hospital of Xinjiang Medical University; 3 Department of Pathology, West China Hospital of Sichuan University, Chengdu, Sichuan 610041, PR China

## Abstract

**Background:**

Cigarette smoking is an important risk factor for pulmonary arterial hypertension (PAH) in chronic obstructive pulmonary disease (COPD). Chymase has been shown to function in the enzymatic production of angiotensin II (AngII) and the activation of transforming growth factor (TGF)-β1 in the cardiovascular system. The aim of this study was to determine the potential role of chymase in cigarette smoke-induced pulmonary artery remodeling and PAH.

**Methods:**

Hamsters were exposed to cigarette smoke; after 4 months, lung morphology and tissue biochemical changes were examined using immunohistochemistry, Western blotting, radioimmunoassay and reverse-transcription polymerase chain reaction.

**Results:**

Our results show that chronic cigarette smoke exposure significantly induced elevation of right ventricular systolic pressures (RVSP) and medial hypertrophy of pulmonary arterioles in hamsters, concurrent with an increase of chymase activity and synthesis in the lung. Elevated Ang II levels and enhanced TGF-β1/Smad signaling activation were also observed in smoke-exposed lungs. Chymase inhibition with chymostatin reduced the cigarette smoke-induced increase in chymase activity and Ang II concentration in the lung, and attenuated the RVSP elevation and the remodeling of pulmonary arterioles. Chymostatin did not affect angiotensin converting enzyme (ACE) activity in hamster lungs.

**Conclusions:**

These results suggest that chronic cigarette smoke exposure can increase chymase activity and expression in hamster lungs. The capability of activated chymase to induce Ang II formation and TGF-β1 signaling may be part of the mechanism for smoking-induced pulmonary vascular remodeling. Thus, our study implies that blockade of chymase might provide benefits to PAH smokers.

## Background

Pulmonary arterial hypertension (PAH) results from a variety of initiating stimuli. Cigarette smoking is an important risk factor for PAH which is frequently developed in patients with severe chronic obstructive pulmonary disease (COPD) [1,2]. The pathogenesis of PAH in smokers is still unclear. In animal models, chronic smoke exposure could cause muscle cell proliferation in small intrapulmonary arteries and induce inflammatory cell influx into the lung, releasing numerous mediators that control the remodeling of pulmonary vessels [3,4].

Chymase, a chymotrypsin-like serine protease which is mainly contained in the secretory granules of the mast cells, has recently been implicated in vascular diseases [5,6]. Like angiotensin-converting enzyme (ACE), chymase is capable of generating angiotensin II (Ang II) from angiotensin I (Ang I). Greater than 80% of Ang II formation in the human heart and greater than 60% in arteries appears to result from chymase activity [7], and chymase-dependent Ang II may have an important role in human cardiovascular system function [8]. Upon stimulations, e.g. vascular injury, mast cells-released chymase can promote vascular proliferation, atherosclerosis, organ remodeling, and tissue fibrosis [6,9]. In monocrotaline-induced PAH rats, Ang II-forming chymase was found to increase pulmonary arteriolar hypertrophy and pulmonary hypertension [10]. Moreover, chymase has recently been reported to induce profibrotic response via transforming growth factor (TGF)-β1/Smad signaling activation [11,12]. Chymase blockade with inhibitors can suppress bleomycin-induced pulmonary fibrosis in hamsters and mice [13,14]. In clinical studies, accumulation of chymase-expressing mast cells is strongly associated with increased vascularity in airway mucosa of asthmatic patients [15]. In smokers, expiratory lung attenuation (Hounsfield units) measured by quantitative computed tomography (CT) analysis correlates negatively with chymase-positive mast cell infiltration in the smooth muscle layer of peripheral airways [16]. In addition, mast cell non-uniform distribution throughout the bronchial tree suggests its involvement in the development of smoking-related peripheral lung injury [17]. However, it still remains unknown whether chymase is involved in cigarette smoke-induced pulmonary artery remodeling and PAH.

The role of chymase in generating Ang II differs among different species. Hamster chymase, like human chymase, is a highly efficient ANG II-forming enzyme [18]. Therefore, in this study, we used hamsters to examine the potential pathophysiological role of chymase in lung vascular remodeling and PAH induced by smoke exposure and to discuss the underlying mechanisms. Our results imply for the first time that chymase may have a role in cigarette smoke-induced pulmonary artery remodeling and pulmonary hypertension in hamsters, possibly through the induction of both Ang II formation and TGF-β1/Smad signaling pathway activation.

## Methods

### Smoke exposure and animal treatment

One-month-old male hamsters, weighing 80-100 g were obtained from the Wu Han Institute of Biological Products (Wu Han, China). All experimental protocols were approved by the Institutional Animal Care and Use Committee of Sichuan University (Chengdu, China).

Hamsters (n = 6/group) were exposed to the whole smoke from 15 commercial nonfilter cigarettes (Wuniu, 14 mg of tar and 1 mg of nicotine per cigarette, Chengdu Cigarette Factor, Chengdu, China) in ventilated whole body exposure chambers (70 × 50 × 50 cm; with a small electric fan inside for chamber mixing) for 30 min each time, twice per day for up to four months with minor modifications as previously described [19]. The smoke total particulate matter (TPM) concentration inside the exposure chambers was 250 ± 26 mg/m3, determined by gravimetric analysis of filters at the exhaust port for the duration of the exposure. Hamsters in control groups were exposed to filtered fresh air under similar conditions.

Chymostatin (1 mg/kg and 2 mg/kg) or distilled saline was administered in a volume of 100 μl by intraperitoneal injection to hamsters 0.5 h before the first smoke exposure each day.

### Hemodynamic analysis

At the end of four months of smoke exposure, right ventricular systolic pressure (RVSP) was recorded. The hamsters were anesthetized with pentobarbitone (50 mg/kg i.p.), and placed in the supine position. An introducer connected to an artery catheter was punctured into the right jugular vein and then into the right ventricle under pressure waveform monitoring. After a period of stabilization, RVSP were recorded using a miniature pressure transducer (Biopac Systems, USA) and a computerized data-acquisition system (MP150; Biopac Systems).

### Histological analysis and tissue preparation

After the animals were sacrificed by carotid artery exsanguination, the left main-stem bronchus was ligated, and 4% polyformaldehyde (pH 7.4) was instilled into the right lung through the trachea under constant pressure (20 cmH_2_O) for 30 min, and then the right lung was removed and submersed in the same fixative overnight at room temperature for paraffin embedding, sectioning and histological staining. The remainder of the lung was dissected, and snap-frozen in liquid nitrogen, then stored at -80°C for biochemical analysis.

The Paraffin sections (4 μm thick) were stained with hematoxylin and eosin (H&E), Masson's trichrome stain and van Gieson's elastic stain. For assessment of vascular morphology, the medial wall thickness (MWT) in fully muscularized arteries with an external diameter of 50 to 100 μm was evaluated by calculating the percentage of medial wall thickness as (medial thickness × 2/external diameter) × 100% along the shortest curvature[10,20]. The external vessel diameter is the distance within external elastic lamella, and the medial thickness is the distance between external and internal elastic laminae. At least 10 muscular arteries per section, 30 arteries per animal were examined using Image Plus 5.0 System (Media Cybernetics, Silver Spring, USA) in a blinded fashion by a skilled investigator.

For the immunohistochemical detection of chymase, the sections were stained with mouse monoclonal antibody to human chymase (1:1000; Chemicon, Temecula, USA) using the VECTASTAIN ABC kit (Vector Laboratories, Burlingame, USA). Preliminary experiments indicated that microwaving for 15 min in 0.01 M citric acid buffer (pH 6.0) [21] was necessary to unmask epitopes for the anti-chymase antibody.

### Measurement of Ang II levels, chymase and ACE activities

Tissue Ang II levels were measured by iodine-125 radioimmunoassay (RIA) using the Ang II RIA kit (Beijing North Institute of Biological Technology, Beijing, China) according to the manufacture's instructions [22]. Briefly, lung tissue was washed with cold saline, minced and heated in 0.1 M HCl at 100°C for 10 min, then homogenized. After centrifugation at 15,000 g for 30 min, the supernatant was lyophilized and redissolved in 400 μl assay buffer, and the radioactivity was measured by a γ counter.

Chymase-like and ACE activities in the lung were also determined by RIA as previously described [22]. Briefly, lung tissue was homogenized in 20 mM cold Tris-HCl (pH 7.4) buffer. Protein concentration was determined using the bicinchoninic acid (BCA) assay (Pierce, Rockford, IL, USA). The serine protease inhibitor aprotinin (Sigma) and the ACE inhibitor lisinopril (Sigma) were used to inhibit proteases other than chymase. The reactions for each sample were divided into three groups, each containing enzyme preparation and 6 ng Ang I as in group one, while 50 μM lisinopril was added in group two and 20 μM aprotinin, 20 mM EDTA plus 50 μM lisinopril were added in group three. A blank control (without sample) was set up for each group. The reaction (total volume 500 μl) was initiated by adding 20 μl of sample followed by incubation at 37°C for 15 min and terminated by adding 2.5 volumes (1.3 ml) of ethanol. After centrifugation at 15 000 g for 30 min, the supernatant was lyophilized and redissolved in the assay buffer provided by the Ang I RIA kit (Beijing North Institute of Biological Technology, Beijing, China) and counted for radioactivity. The enzymatic activities were determined based on the decrease of Ang I. One unit (U) of activity was defined as the amount of enzyme producing 1 ng Ang I decrease per min. The activity not inhibited in the presence of lisinopril, aprotinin and EDTA was considered to be chymase-like activity and the activity inhibited by lisinopril was considered to be ACE activity.

### RNA Isolation and reverse-transcription polymerase chain reaction (RT-PCR) analysis

Total RNA was isolated using Trizol (Invitrogen, Carlsbad, CA, USA) from the frozen tissue. First-strand cDNA was synthesized from 5 μg of total RNA for each sample using MMLV reverse transcriptase (MBI Fermentas Inc, Ontario, Canada) and random hexamer primers, according to the manufacturer's instructions. Primers for chymase PCR (738 bp) were (forward) 5'-CTG AGA GGA TGC TTC TTC CTG C-3' and (reverse) 5'-AGA TCT TAT TGA TCC AGG GCC G-3' [23]. Primers for β-actin PCR (194 bp) were (forward) 5'-CCT GTA TGC CTC TGG TCG TAC C-3' and (reverse) 5'-TCT CGG CTG TGG TGG TGA AG-3'. The PCR program for chymase was initiated by a 2 min denaturation step at 94°C, followed by 35 cycles of 94°C for 30 s, 63°C for 30 s and 72°C for 1 min, and a final extension at 72°C for 5 min. PCR products were electrophoresed on a 1.5% agarose gel and visualized by ethidium bromide staining. Densitometry was carried out using a Bio-Rad ChemiDoc image acquisition system and Quantity One (v4.6) quantitation software (Bio-Rad, Hercules, CA, USA).

### Western blotting analysis

Lung homogenates were prepared in lysis buffer, containing 50 mM Tris-HCl, 150 mM NaCl, 1% NP-40, 0.5% sodium deoxycholate, 2 mM NaF, 2 mM EDTA, 0.1% SDS, and a protease inhibitor cocktail tablet (Roche Applied Science, Indianapolis, USA). Equivalent amounts of protein (30 μg) from each sample were separated on 10% SDS-polyacrylamide gels, and then transferred onto 0.45 μM polyvinylidene difluoride (PVDF) membranes (Millipore, Bedford, USA). Primary antibodies used were chymase monoclonal antibody (1:1000; Chemicon, Temecula, USA), TGF-β1 polyclonal antibody (1:500; Santa Cruz Biotechnology, Santa Cruz, USA), Smad2/p-Smad2 polyclonal antibody (1:1000; Cell Signaling, Beverly, USA), Smad3/p-Smad3 polyclonal antibody (1:1000; Cell Signaling, Beverly, USA). The signals were developed using Super-Signal West Pico chemiluminescent substrate (Pierce, Rockford, USA).

### Statistical analyses

Values were expressed as mean ± SD. Statistical analysis was carried out using one-way ANOVA, followed by Tukey's HSD test for *post hoc *multiple comparisons (SPSS for Windows version 13.0, Chicago, USA). A significant difference was accepted at *P *< 0.05.

## Results

### Chronic cigarette smoke exposure leads to pulmonary artery remodeling and pulmonary hypertension in hamsters

**Figure 1:**
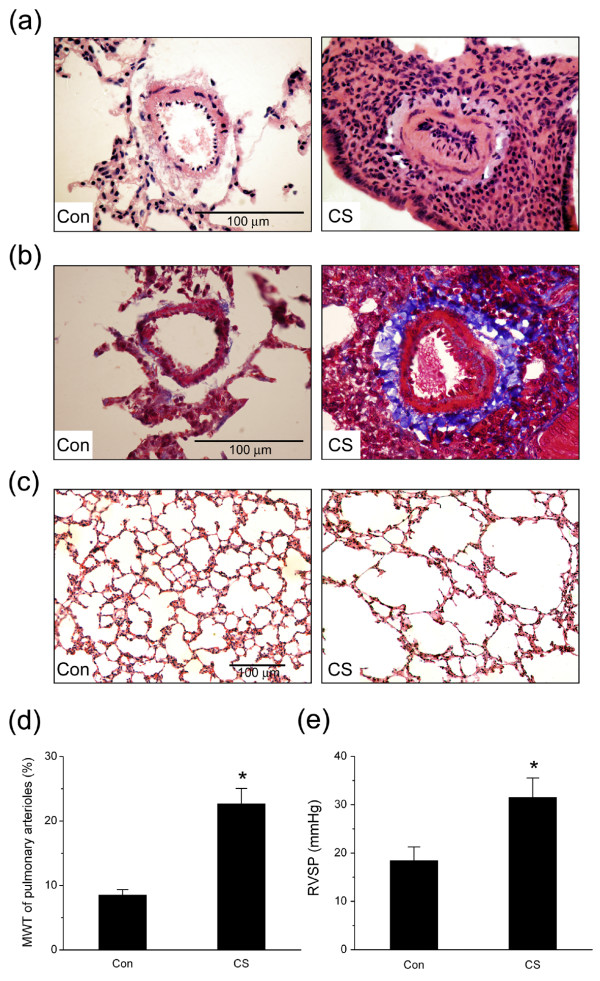
**Cigarette smoke-induced changes in pulmonary vascular and alveolar morphology and right ventricular systolic pressure (RVSP)**. (a) Representative hematoxylin and eosin (H&E) staining of small pulmonary vessels (original magnification × 40). (b) Representative Masson's trichrome staining of small pulmonary vessels (original magnification × 40). (c) Emphysema-like lesions in the lung after smoke exposure (H&E staining, original magnification × 20). (d) Medial wall thickness (MWT) of pulmonary arterioles. (e) RVSP in hamsters. Con: control group; CS: cigarette smoke-exposed group. Scale bars = 100 μm. Values are expressed as mean ± SD (n = 6). * *P *< 0.05, significant difference from the control group.

After four months of cigarette smoke exposure, thick-walled pulmonary arterioles with inflammatory cell infiltration, intima hyperplasia, vascular smooth muscle hypertrophy and deposition of collagen around vessel wall were observed in the smoke-exposed hamster lungs compared to the normal vascular structure in the control hamster lungs (Fig. 1a, H&E stain; Fig. 1b, Masson's trichrome stain). Concurrently, hamsters developed emphysema-like airspace enlargement in lung periphery after 4 months of cigarette smoke exposure (Fig. 1c).

The medial wall thickness (MWT) of the arterioles, which is an index of pulmonary artery remodeling, was significantly increased after cigarette smoke exposure (Fig. 1d; n = 6, *P *< 0.05). The changes in pulmonary artery pressure were assessed by measuring RVSP via right heart catheterization. In the smoke-exposed group, RVSP was significantly higher than in the control group (31.50 ± 4.02 vs. 20.42 ± 1.54 mmHg; Fig. 1e, n = 6, *P *< 0.05).

### Up-regulation of chymase expression in smoke-exposed lungs

**Figure 2:**
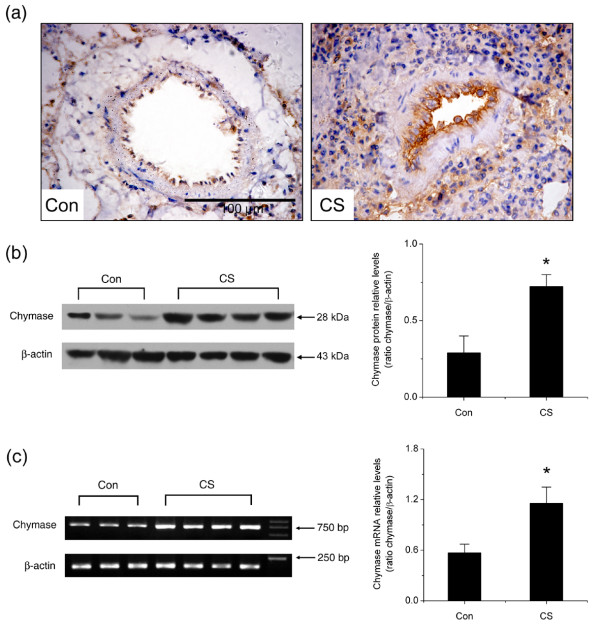
**Changes in chymase protein and mRNA levels in hamster lungs**. (a) Representative chymase immunohistochemical staining in pulmonary arterioles (original magnification × 40). (b) Representative Western blotting analysis of chymase protein levels in hamster lungs. (c) Representative RT-PCR analysis of chymase mRNA levels in hamster lungs. Con: control group; CS: cigarette smoke-exposed group. Scale bars = 100 μm. Data are expressed as mean ± SD (n = 3 for control group and n = 4 for smoke-exposed group). * *P *< 0.05, significant difference from the control group.

To determine whether chymase is involved in cigarette smoking-induced pulmonary artery remodeling and PAH, chymase protein and mRNA levels in the lungs of the smoke-exposed hamsters and the control hamsters were compared. Immunohistochemical analysis revealed notable increase of chymase positive staining area in the adventitia and hyperplastic intima of pulmonary arterioles in the smoke-exposed hamster lungs as compared with the control lungs (Fig. 2a). Furthermore, Western blotting results showed that the relative protein levels for chymase in smoke-exposed lung homogenates were nearly 2.5 fold higher than in the control ones (Fig. 2b, *P *< 0.05). To further examine chymase gene expression at transcriptional level, the steady-state mRNA levels for chymase and β-actin in lung tissue were analyzed by RT-PCR. The accumulation of chymase mRNA in hamster lungs was also significantly induced by cigarette smoke exposure (Fig. 2c, *P *< 0.05). Together, these results suggested that chymase expression was up-regulated in cigarette smoke-exposed hamster lungs at both mRNA and protein levels.

### Increase in chymase-like activity in the lung after chronic cigarette smoke exposure

**Figure 3:**
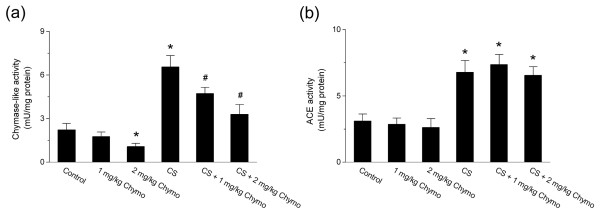
**Changes of chymase-like and ACE activities after chymase inhibition with chymostatin in hamster lungs**. (a) Chymase-like activity. (b) ACE activity. Control: control group; CS: cigarette smoke-exposed group; 1 mg/kg Chymo: hamsters treated with 1 mg/kg chymostatin alone; 2 mg/kg Chymo: hamsters treated with 2 mg/kg chymostatin alone; CS + 1 mg/kg Chymo: hamsters treated with cigarette smoke plus 1 mg/kg Chymostatin; CS + 2 mg/kg Chymo: hamsters treated with cigarette smoke plus 2 mg/kg Chymostatin. Values are expressed as mean ± SD (n = 6). * *P *< 0.05, significant difference from the control group. # *P *< 0.05, significant difference from the smoke-exposed group.

Since chymase expression in the lung was up-regulated by cigarette smoke exposure, we then measured the changes in chymase-like and ACE activities in lung homogenates. Results showed that both chymase-like and ACE activities were increased by smoke exposure (Fig. 3; n = 6, *P *< 0.05). The chymase inhibitor, chymostatin, significantly reduced the cigarette smoke-induced increase in chymase-like activity, whereas it had no effect on ACE activity (Fig. 3; n = 6, *P *< 0.05).

### Chymase inhibition attenuated cigarette smoke-induced pulmonary artery remodeling and pulmonary hypertension

**Figure 4:**
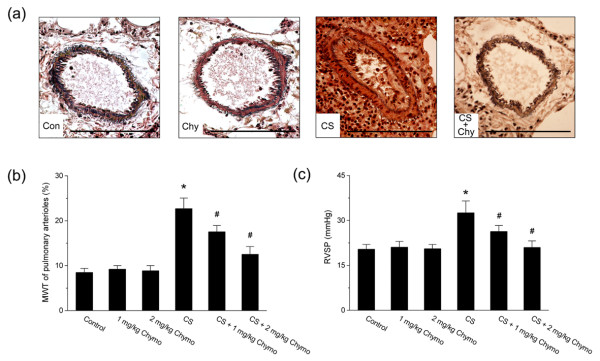
**Changes in the remodeling of pulmonary arterioles and RVSP after chymase inhibition with chymostatin**. (a) Representative van Gieson's elastic staining of small pulmonary vessels (original magnification × 40). Scale bars = 100 μm. Con: control; CS: cigarette smoke exposure; Chy: treatment with 2 mg/kg chymostatin alone; CS+Chy: treatment with smoke exposure plus 2 mg/kg chymostatin. (b) MWT of pulmonary arterioles. (c) RVSP. Control: control group; CS: cigarette smoke-exposed group; Chymo: chymostatin treatment. Values are expressed as mean ± SD (n = 6). * *P *< 0.05, significant difference from the control group. # *P *< 0.05, significant difference from the smoke-exposed group.

As compared with hamsters exposed to cigarette smoke alone, the hamsters exposed to cigarette smoke plus 1 mg/kg or 2 mg/kg chymostatin pre-administration showed an attenuated induction of pulmonary artery remodeling as indicated by MWT index (Fig. 4a, b; n = 6, *P *< 0.05). Similarly, chymostatin treatment also significantly inhibited the cigarette smoke-induced increase in RVSP (Fig. 4c; n = 6, *P *< 0.05).

### Chymase inhibition reduced cigarette smoke-induced Ang II accumulation and TGF-β1/Smad signaling activation

**Figure 5:**
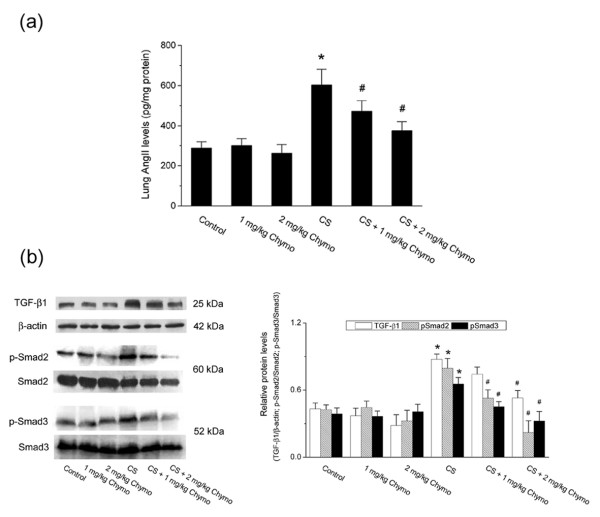
**Changes in Ang II levels and TGF-β1/Smad signaling activation in hamster lungs**. (a) Ang II levels. Values are expressed as mean ± SD (n = 6). (b) Protein levels of TGF-β1, β-actin, p-Smad2, Smad2, p-Smad3 and Smad3 measured by Western blotting analysis. Images are representative of three independent experiments. Relative protein levels were assessed by densitometry. Control: control group; CS: cigarette smoke-exposed group; Chymo: chymostatin treatment. * *P *< 0.05, significant difference from the control group. # *P *< 0.05, significant difference from the smoke-exposed group.

To determine the involvement of chymase pathway in cigarette smoke-induced pulmonary hypertension, we assessed Ang II concentration and TGF-β 1/Smad signaling activation in hamster lungs. In the smoke-exposed group, lung Ang II levels were significantly higher than in the control group (602.17 ± 79.41 vs. 287.93 ± 31.25 pg/mg protein). Both 1 mg/kg and 2 mg/kg chymostatin treatment significantly decreased the lung tissue Ang II concentration as compared with the smoke-exposed group (Fig. 5a; n = 6, *P *< 0.05).

TGF-β1, Smad2/p-Smad2, and Smad3/p-Smad3 were detected by Western blotting analysis. Results showed that TGF-β1, p-Smad2, and p-Smad3 protein levels were markedly increased in the smoke-exposed lungs compared to the control lungs (Fig. 5b). Blockade of chymase with chymostatin resulted in a significant reduction of TGF-β1, p-Smad2 and p-Smad3 as compared with the smoke-exposed group. In contrast, chymostatin exerted no inhibitory effects on the total Smad2 and Smad3 protein levels. Taken together, these results suggest that cigarette smoke exposure causes enhanced Ang II accumulation and activation of TGF-β1/Smad signaling pathway, which could be suppressed by chymase inhibition with chymostatin.

## Discussion

In this study, we demonstrated the potential role of chymase in cigarette smoke-induced pulmonary artery remodeling and pulmonary arterial hypertension. In the hamster model studied here, chronic smoke exposure induced intima proliferation, smooth muscle hypertrophy and collagen deposition in pulmonary arterioles, which may lead to increased RVSP. Our results indicated that chronic cigarette smoke exposure significantly increased chymase synthesis and activity in the lung, and that chymase inhibition with chymostatin effectively attenuated the smoke-induced pathophysiological changes in pulmonary arterioles, possibly through inhibiting the conversion of AngII and the activation of TGF-β1/Smad signaling pathway.

Numerous studies have reported that chymase, acting as an important component of the local renin-angiotensin system (RAS), is activated in vascular disease conditions, such as hypertension and atherosclerosis [24,25]. High levels of chymase have been found in both spontaneously hypertensive rats and monocrotaline-induced PAH rats [7,10]. Cigarette smoking is a major risk factor for pulmonary airway and vascular diseases [26,27]. In smokers, mast cells containing chymase in peripheral airways may contribute to the relationship between air trapping and airway inflammation [16]. In in vitro studies, mast cells exposed to cigarette smoke condensate revealed a marked increase in chymase transcript levels [28]. In the present study, we found that chronic cigarette smoke exposure significantly up-regulated chymase expression at both mRNA and protein levels in hamster lungs, which was associated with increased artery remodeling, emphysema-like changes and RVSP elevation. ACE and chymase-like activities in the lung were also increased in response to cigarette smoke exposure. As indicated by chymase immunohistochemistry, chymase-containing mast cell accumulation and chymase release into the interstitial lung tissue might contribute to the increase of chymase expression in hamster lungs.

Given the potential role of chymase in pulmonary hypertension, we sought to test whether the well-studied chymase inhibitor chymostatin can reverse the damaging effects of cigarette smoke. The results showed that chymostatin administration significantly reduced the smoke-induced increase in chymase activity but had no effect on ACE activity, suggesting that chymostatin was mainly acting through the inhibition of chymase in the lung. Recent studies have demonstrated that chymase plays a functional role in ACE-independent generation of AngII, which occurs immediately after its release into the interstitial tissues after vascular injury [5,9,29]. In our results we could show a two fold increase in lung AngII levels after four months of cigarette smoke exposure, which was significantly reduced by chymase inhibition with chymostatin. Chymostatin treatment also reduced pulmonary arteriolar hypertrophy and RVSP as compared with the smoke-exposed hamster lungs. These results indicate that chymase might be an alternative pathway for local pulmonary AngII formation and play an important role in the cigarette smoke-induced PAH.

The roles for chymase in disease progression are not limited to AngII generation; chymase also has widespread effects independent of AngII formation, including activation of TGF-β1 and angiogenesis [14,15]. Previous studies have reported that chymase activates latent TGF-β to form mature TGF-β and increase collagen production [30,31]. The active form of TGF-β exerts many biological actions in the pathogenesis of lung disease, including the stimulation of fibroblast proliferation, extracellular collagen deposition, cell proliferation, and angiogenesis [32,33]. Smads are the major transducer of TGF-β signaling pathway in lung fibrosis. Mice exposed to cigarette smoke up to 6 months showed increased p-Smad2 protein levels in the lung, indicating enhanced TGF-β downstream signaling by smoke exposure [34]. Chymase has recently been reported to activate the TGF-β1/Smad signaling pathway in rat cardiac fibroblasts [11]. Chymase inhibition can decrease TGF-β1 transcription levels and prevent cardiac fibrosis in animal models [35,36]. In the present study, TGF-β1, p-Smad2, p-Smad3 protein levels are markedly up-regulated in the smoke-exposed hamster lungs, which could be all reduced by chymase blockade with chymostatin. These results imply that chymase activation and upregulation by chronic smoke exposure might also enhance the TGF-β1/Smad signaling pathway and promote pulmonary artery remodeling in hamster lungs.

One limitation of this study is that chymostatin is known to inhibit other serine proteases, such as cathepsin G which is also capable of generating Ang II from Ang I [37] and cathepsin D which can also activate latent TGF-β1 [38]. So the use of chymostatin cannot unequivocally indicate the relative contribution of different serine proteases in the generation of Ang II and the activation of TGF-β1. Nevertheless, as established in literature, chymostatin was found to be much more potent as inhibitors of chymase than of cathepsin G and cathepsin D activities [39,40]. Furthermore, chymase is the predominate enzyme for ACE-independent production of Ang II in vascular tissues of humans, monkeys, dogs, and hamsters [41,42]. Thus our results are likely to reflect roles of chymase in cigarette smoke-induced PAH in hamsters.

## Conclusions

In summary, our data reveal that chymase activity and expression was significantly increased in chronic cigarette smoke-induced pulmonary hypertensive hamsters with elevated RVSP and remodeling of pulmonary arterioles. In addition, chymase inhibition with chymostatin significantly decreased not only RVSP but also AngII levels and TGF-β1/Smad signaling pathway activation in smoke-exposed lungs. These results suggest that the capability of activated chymase to induce Ang II formation and TGF-β1 signaling may be part of the mechanism for smoking-induced pulmonary vascular remodeling. Thus, our study implies that blockade of chymase might provide benefits to PAH smokers.

## Abbreviations

ACE: angiotensin converting enzyme; Ang I: angiotensin I; Ang II: angiotensin II; COPD: chronic obstructive pulmonary disease; MWT: medial wall thickness; PAH: pulmonary arterial hypertension; RVSP: right ventricular systolic pressures; TGF-β1: transforming growth factor-β1

## Competing interests

The authors declare that they have no competing interests.

## Authors' contributions

TW and SXH designed the experiment, carried out the data analysis and drafted the manuscript. SXH, LC, YYN and DX carried out the animal experiment. SFZ did the histopathological analysis. TW, YJC, GMH, JA and XRH carried out the RT-PCR, Western blot, and enzymatic activity assays. XHZ and FQW participated in the conception, design and coordination of the studies and critically reviewed the manuscript. All authors have read and approved the final manuscript.
